# Effects of Modulated Electro-Hyperthermia (mEHT) on Two and Three Year Survival of Locally Advanced Cervical Cancer Patients

**DOI:** 10.3390/cancers14030656

**Published:** 2022-01-27

**Authors:** Carrie Anne Minnaar, Innocent Maposa, Jeffrey Allan Kotzen, Ans Baeyens

**Affiliations:** 1 Department of Radiation Sciences, University of the Witwatersrand, Johannesburg 2193, South Africa; carrie-anne.minnaar@wits.ac.za (C.A.M.); jeffrey.kotzen@wits.ac.za (J.A.K.); 2 Department of Radiation Oncology, Wits Donald Gordon Academic Hospital, Johannesburg 2193, South Africa; 3 Department of Epidemiology & Biostatistics, University of the Witwatersrand, Johannesburg 2193, South Africa; innocent.maposa@wits.ac.za; 4 Radiobiology, Department of Human Structure and Repair, Ghent University, 9000 Ghent, Belgium

**Keywords:** modulated electro-hyperthermia, abscopal effect, locally advanced cervical cancer, resource-constrained setting, radiosensitiser

## Abstract

**Simple Summary:**

More than 80% of global cervical cancer cases and deaths occur in Low-to-Middle-Income Countries. Improving the efficacy of treatments without increasing the costs in these regions is therefore imperative. The aim of our Phase III Randomised Controlled Trial was to investigate the effects of the addition of a mild heating technology, modulated electro-hyperthermia, to chemoradiotherapy protocols for the management of locally advanced cervical cancer patients in a resource-constrained setting. We previously reported on the positive outcomes on local disease control, quality of life, and early toxicity. Our recent results showed a significant improvement in two and three year disease free survival, without any significant changes to the toxicity profile, and with an improvement in quality of life, alongside a cost saving over three years. The effect was most significant in patients with Stage III disease, and a significant systemic effect was observed in patients with distant nodal metastases.

**Abstract:**

(1) Background: Modulated electro-hyperthermia (mEHT) is a mild to moderate, capacitive-coupled heating technology that uses amplitude modulation to enhance the cell-killing effects of the treatment. We present three year survival results and a cost effectiveness analysis from an ongoing randomised controlled Phase III trial involving 210 participants evaluating chemoradiotherapy (CRT) with/without mEHT, for the management of locally advanced cervical cancer (LACC) in a resource constrained setting (Ethics Approval: M120477/M704133; ClinicalTrials.gov ID: NCT033320690). (2) Methods: We report hazard ratios (HR); odds ratio (OR), and 95% confidence intervals (CI) for overall survival and disease free survival (DFS) at two and three years in the ongoing study. Late toxicity, quality of life (QoL), and a cost effectiveness analysis (CEA) using a Markov model are also reported. (3) Results: Disease recurrence at two and three years was significantly reduced by mEHT (HR: 0.67, 95%CI: 0.48–0.93, *p* = 0.017; and HR: 0.70, 95%CI: 0.51–0.98, *p* = 0.035; respectively). There were no significant differences in late toxicity between the groups, and QoL was significantly improved in the mEHT group. In the CEA, mEHT + CRT dominated the model over CRT alone. (4) Conclusions: CRT combined with mEHT improves QoL and DFS rates, and lowers treatment costs, without increasing toxicity in LACC patients, even in resource-constrained settings.

## 1. Introduction

Around 602,127 new cases of cervical cancer and an estimated 341,831 deaths from cervical cancer were reported globally in 2020. More than 80% of these cases and deaths occurred in Low-to-Middle-Income-Countries (LMICs) [1], creating significant socio-economic stress in these resource-constrained settings [2]. The problem is compounded by poor screening programs [2], limited access to adequate treatments [3], and the high incidence of Human Immunodeficiency Virus (HIV) infections in these regions [4]. While developed countries are estimated to achieve the elimination goal of four cases per 100,000 women-years by 2060, LMICs are expected to only reach this goal towards the end of the century [2]. Improving treatment outcomes, without significantly increasing the costs, is therefore crucial to the management of the disease in these regions. Hyperthermia (HT) is a known radiosensitiser [5], and has proven to be a beneficial adjunct to radiotherapy (RT) and chemoradiotherapy (CRT) for the management for locally advanced cervical cancer (LACC) in developed settings [6]. Classical HT techniques include capacitive and radiative heating technologies, both of which have demonstrated efficacy at improving outcomes in cervical cancer [7,8,9]. Classical HT uses temperature-dependent dosing calculations such as CEM43 and TRISE [10,11] to optimise the treatment outcomes, although the optimal temperature and timing is still a topic of discussion [12,13].

There is emerging evidence that radiofrequency (RF) electromagnetic fields associated with some HT techniques have additional effects during the treatments [14]. Modulated electro-hyperthermia (mEHT) is a mild- to moderate-heating technology that applies 13.56 MHz RF waves generated by a capacitive coupling set-up between two electrodes. The amplitude of the waves is modulated with a signal equivalent to 1/f noise, where the power density (S(f)), (or power per frequency interval), of the 1/f amplitude-modulated signal is inversely proportional to the modulation signal: S(f)~1/f. The amplitude modulation (AM), and the precise impedance matching (which allows for the cellular selection and the relatively low applied power), are the main differences between mEHT and classical capacitive HT technologies [15]. Pre-clinical studies have shown that the modulation induces a non-thermal field effect which enhances the cell–killing of the thermal effect by a factor of 3.2 [16]. This appears to make mEHT more effective when adjusted to the same temperature as other heating techniques in pre-clinical studies [17]. It has even been proposed that the AM could be the most important characteristic of mEHT [18]. Pre-clinical studies have shown several immune-related effects of mEHT, which, if applied clinically, could promote the recognition and the targeting of tumours by the immune system [19,20,21,22].

This technique proposes a dosing paradigm based on energy deposition and absorption, with thermal effects being an outcome of the treatment, and not the goal of the treatment. The biophysics of the technology are described in detail elsewhere in the literature [23,24]. The lower power output, lower temperatures achieved [25], and non-thermal dosing parameters negate the need for thermal monitoring as safety and dosing parameters during mEHT. This has led to opposing opinions regarding the grouping of mEHT with classical HT techniques.

While there are numerous Phase I/II trials on mEHT, and some small double arm studies [26], there have not been any completed Phase III Randomised Controlled Trials (RCT) on mEHT. We previously reported preliminary results from an ongoing Phase III RCT which is investigating the effects of CRT with or without mEHT for the management of LACC in a resource-constrained setting in South Africa. The primary outcome was two year overall survival (OS), and the secondary outcome was local disease control (LDC) at six months post-treatment. The LDC results, as summarised in Table 1 [27], and a detailed safety and toxicity analysis [28], have been reported previously. The Odds Ratios (OR) for achieving LDC and Local Disease Free Survival (LDFS) at six months post-treatment were 0.39 (95%CI: 0.20–0.77; *p =* 0.006) and 0.36 (95%CI: 0.19–0.69; *p* = 0.002), respectively, in favour of the administration of mEHT [27].

The addition of mEHT did not affect the early toxicity profile of the prescribed CRT. In the mEHT Group, 97% of the participants were able to receive ≥ 8 out of the 10 prescribed mEHT treatments, with 9.5% of participants in the mEHT group reporting grade 1–2 adipose burns, 2% reporting grade 1 surface burns, and 8.6% reporting pain during the mEHT treatments [28]. The average BMI of the participants was 27.8 [15,16,17,18,19,20,21,22,23,24,25,26,27,28,29,30,31,32,33,34,35,36,37,38,39,40,41,42,43,44,45,46,47,48,49]. A multivariate analysis showed that energy dose in kilojoules, HIV status, and Body Mass Index (BMI) were not significant predictors of adverse events. Body Mass Index was also not significantly predictive of LDC. This suggests that mEHT is able to penetrate thicker layers of adipose tissue than conventional capacitive heating technologies, without significant damage to the adipose tissue [28]. The addition of mEHT was also associated with a significantly greater improvement in cognitive function at six weeks post-treatment, a significant reduction in pain and fatigue, and a significant improvement in social and emotional functioning at three months post-treatment [28]. An unexpected observation was the potentiation of the abscopal effect. An analysis of the sub-group of participants with extra-pelvic nodal disease visualised on the pre-treatment 18^F^-FDG PET/CT scans showed that 24.1% (13 out of 54) of those who were treated with mEHT had complete metabolic resolution of all disease on the follow-up 18^F^-FDG PET/CT scans, compared to 5.6% (3 out of 54) of the participants who did not receive mEHT (Chi squared: *p* = 0.013). A multivariate analysis showed that the outcomes were not associated with the administration of cisplatin or with the participants’ HIV-status. These results suggested the potentiation of an abscopal effect by mEHT, as the locally applied RT resulted in the resolution of distant disease, when combined with mEHT. These findings are elaborated in the paper by Minnaar et al. [29].

The preliminary results showed a significant short-term benefit with the addition of mEHT to CRT, without a significant increase in toxicity, in our resource-constrained setting. We present the two and three year OS results, and preliminary results from a cost effectiveness analysis (CEA) on the use of mEHT in public and private healthcare settings. Local disease control may be associated with a short-term improvement in quality of life and with OS; however, long-term DFS results hold more relevance as DFS may be associated with sustained improvements in quality of life and affect the socio-economic impact of the disease. The follow-up results presented in this paper are the first long-term results reported from a Phase III RCT on mEHT and they are an important contribution to the understanding of the long-term clinical impact of mEHT in the management of LACC. The CEA provides valuable insight into the feasibility of incorporating mEHT into clinical practice that can be applied to both developed and resource-constrained settings.

## 2. Materials and Methods

The trial (ClincialTrials.gov ID: NCT03332069), was approved by the Human Research and Ethics Committee (HREC) on 4 May 2012 (ID: M120477) and registered on the National Clinical Trial Database (ID: 3012) before recruitment began. Due to the significant improvement seen in the mEHT Group early on in the study, the follow-up period was extended from two to five years post treatment on 5 May 2017 (M704133). All patients (or their legal representatives) provided written informed consent before enrolment.

Participants: Inclusion criteria included females with treatment-naïve, histologically confirmed FIGO stage IIB (with invasion of the distal half of the parametrium) to IIIB squamous cell carcinoma of the uterine cervix (staged clinically using a chest X-ray, abdomino-pelvic ultrasound, and clinical examination); eligible for RT with radical intent; and a creatinine clearance > 60 mL/min (calculated according to the Cockcroft-Gault equation). Additional inclusion criteria included an Eastern Cooperative Oncology Group (ECOG) performance status < 2; estimated life expectancy of at least 12 months; adequate haematological function (absolute neutrophil count > 3000/mm^3^, haemoglobin ≥ 10 g/dL; platelet count > 150/mm^3^); and a negative pregnancy test and use of effective contraception in women of childbearing potential. Pre-treatment 18^F^-FDG PET/CT scans were performed as part of the screening process. Patients with Vesicovaginal and vesicorectal fistulas; extra-pelvic visceral metastases, and bilateral hydronephrosis visualised on screening 18^F^-FDG PET/CT scans, were excluded from the study, as were HIV-positive patients with a CD4 count < 200 cells/µL and/or not on antiretroviral therapy (ART) for at least six months and/or signs of ART resistance; contradictions or a known hypersensitivity to any of the prescribed treatments; life-threatening Acquired Immunodeficiency Syndrome (AIDS) defining illnesses (other than cervical carcinoma); prior invasive malignancy, other than LACC, diagnosed within the past 24 months; and pregnant or breast feeding women. For the analyses in this report, all participants who met the eligibility criteria, were randomised, were treated, and for whom data were available at two years and three years post-treatment, were included. Participants who were lost to follow-up are reported as “LTFU” and their last known disease status is included.

Treatment: As per institutional protocols, all participants received 50 Gy of external beam radiotherapy (EBRT) in 25 fractions, administered to the whole pelvis, using 2D planning with virtual simulation. High Dose Rate (HDR) brachytherapy (BT) (source used: Iridium-192), was administered in three fractions of 8 Gy for a total equivalent dose in 2 Gy fractions (for an alpha-beta ratio of 10) of 86 Gy. Further details of the RT method can be found in the paper by Minnaar et al. [27]. 2D planning for EBRT and HDR BT is standard in our facility and in resource-constrained settings due to the lack of access to sophisticated imaging techniques and due to limited resources and staff capacity available to manage the high volume of gynaecological oncology patients seen each year. All participants were prescribed two doses of 80 mg/m^2^ cisplatin, administered 21 days apart (according to the institutional protocol), during EBRT (not administered on BT days or mEHT days). Participants in the study group received two mEHT treatments per week (Model: EHY2000+; Manufacturer: Oncotherm GmbH, Troisdorf, Germany), with a minimum of 48 h in between mEHT treatments, at a target power of 130 W for a minimum of 55 min. The EBRT was started within thirty minutes of completing mEHT treatments. Total Kilojoules administered per treatment were recorded.

Randomisation and Masking: After enrolment, participants were randomly assigned (stratum: HIV status; accounting for age and stage), to receive CRT alone, or in combination with mEHT, using the REDCap stratified secure online random-sampling tool. Although the trial was open-label, and participants were aware of which group they were in due to the challenges associated with setting up a sham hyperthermia treatment, physicians reporting on the pre- and six month post-treatment 18^F^-FDG PET/CT scans were blinded to the group that the participants were in, as were the clinicians conducting the follow-up evaluations.

Data Collection and Management: The research co-ordinator was responsible for collecting the data and data were captured using the online REDCap electronic data capture tool hosted by the University of the Witwatersrand. The treatments were administered and the clinical evaluations were conducted by the clinical team, without the involvement of the research co-ordinator.

Outcomes: The primary outcome was Two Year OS. Two year DFS, defined as the time from the start of treatment until the time of first documented disease recurrence, is also reported. The first evaluation of LDC was done at six months post-treatment. If local disease was still visible on the six month 18^F^-FDG PET/CT, then DFS was considered a failure from day one and the number of days spent disease free was considered to be zero. Three year OS and DFS are also reported. The DFS was censored for cancer specific deaths. Participants who demised with a disease free status, and who did not demise from a treatment related death, before the two or three year cut off, were allocated a positive disease free status at the exit date and the exit date was recorded as the date of death. Late toxicity was graded according the Common Toxicity Criteria for Adverse Events (CTCAE) version 4, and Quality of life (QoL) was measured using the validated European Organization for Research and Treatment of Cancer (EORTC) quality of life questionnaires (QLQ): C30 and Cx24 (cervical cancer specific). The QLQs were available in several local languages [30] and were administered at one and two years post-treatment. The results were compared to the baseline QLQ results and the scoring and reporting were done in accordance with the EORTC guidelines [31,32]. According to the EORTC guidelines, the scores were converted linearly to scores from 0–100, where a high score represents higher functioning or a higher symptom experience, and a lower score represents a lower symptom experience or a lower functioning [33]. Early toxicity and QoL at six months post treatment have been previously reported [27,28]. A CEA was performed, and the outcome was Cost per Quality Adjusted Life Year (QALY). After initial treatment costs, only disease progression and hospitalization costs are further incurred in the model. The private healthcare model included costs associated with Intensity-Modulated Radiation Therapy (IMRT), weekly cisplatin and a broader range of chemotherapy drugs for recurrent or residual disease, whereas the public healthcare model included only 3D-planning for radiotherapy, two doses of cisplatin during RT, and limited treatment options for recurrent or residual disease.

Statistical Analysis: The sample size was calculated based on the estimated required sample sizes for a two-sample comparison of survivors’ functions at two years (statistical power of 90%). We estimated an expected reduction in mortality at two years of 50%, based on OS of 20% in the Control Group and 40% in the experimental group. The statistical significance is defined as a two-sided alpha < 0.05 for a log-rank test, with a constant Hazard Ratio (HR) of 0.5693. Cox proportional hazards models including each factor (treatment group, HIV status, age, stage of the disease) were performed to compare the time from the start of treatment to the first occurrence of any event (death or disease recurrence). We report the HR; Odds Ratio (OR), and 95% confidence interval. Log-rank statistics were used to compare both treatment arms with Kaplan–Meier survival curves plotted at two and three years (for OS; DFS), for stage IIB and stage III participants separately and combined. Overall type I error was considered at 5%, and the survival analysis was done by intention to treat. The initial survival analysis was planned for two years post-treatment. However, the positive results seen at two years post-treatment motivated an extension of the follow-up to five years post-treatment. In this paper, we therefore include the original planned two year analysis as well as the three year analysis, which was used for the evaluation of the cost effectiveness. Late toxicity was graded according to the CTCAE V.4 for bone, renal, bladder, skin, subcutaneous tissues, mucous membranes and gastrointestinal systems. The frequency of reported grade 1/2 late toxicity and grade 3/4 toxicity were compared by treatment group and by HIV status using frequency tables. Pearson’s Chi squared test and Fisher’s exact tests were used to determine the difference in frequencies between groups. Multivariable proportional hazards regression models were used to identify significant predictors (including arm, HIV status, and number of cisplatin doses), of grades 3/4 late toxicity. Two-sample independent *t*-tests with equal variances were used to evaluate QoL score change from baseline to 12 and 24 months post-treatment between the two treatment groups. The differences in score changes between the groups are assessed using paired *t*-tests. STATA 15.0 Statistics software program (Stata Corporation, College Station, TX, USA) was used to analyse the data.

The CEA was performed with a time horizon of three years, using a Markov model with a six month cycle length, from the perspective of a private healthcare funder (medical aid scheme), and a public healthcare funder (the state). The two-tiered healthcare system in South Africa is comprised of a state-funded public healthcare system and a private healthcare system that is mostly funded by private contributions to medical aid schemes. An estimated 70–80% of the population makes use of the public healthcare system [34], and this setting is underfunded and poorly equipped to manage the large volume of patients. The input costs of the treatments for the public healthcare CEA are based on the direct costs to the state for the treatments, as outlined in by the Department of Health [35], and therefore represents the cost versus benefit of the treatment of patients in a public healthcare facility, funded by the state. The input costs of the treatments in the private healthcare CEA include the regulated profit added to the cost of the treatments, charged by the privately owned hospitals and by the healthcare professionals in private practice, to the private medical aid schemes. The results of the private healthcare CEA therefore represent the costs to the private healthcare funders, versus the clinical benefit of the members or patients.

## 3. Results

### 3.1. Participants

**Figure 1:**
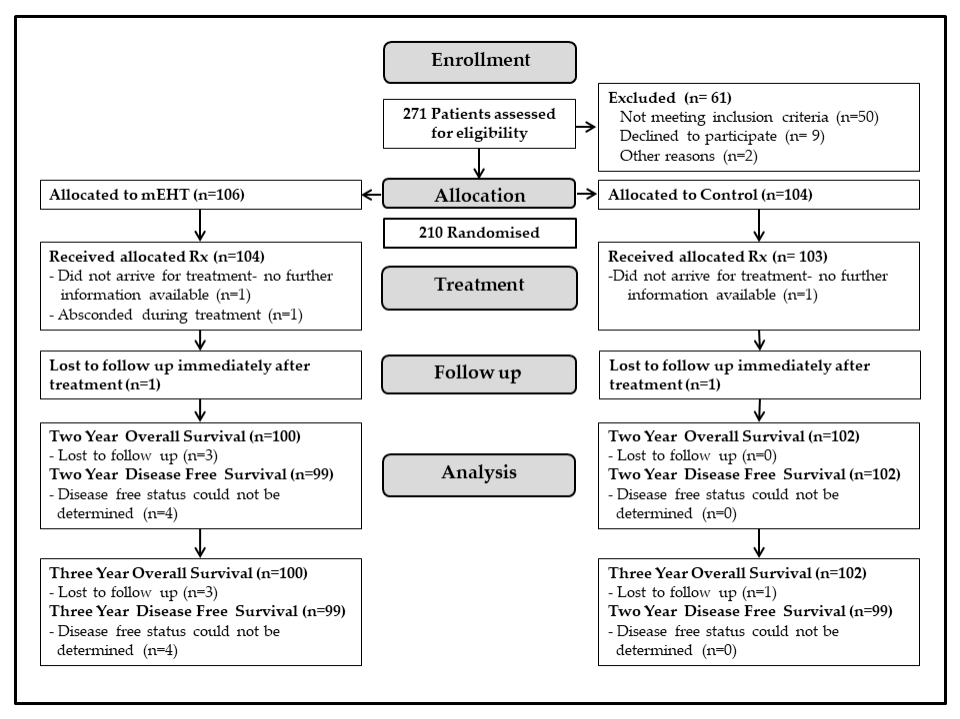
Trial profile. Abbreviations: mEHT: modulated electro-hyperthermia.

A total of 271 patients were screened between January 2014 and November 2017, and 210 eligible participants were enrolled and randomised (mEHT Group: *n* = 106, Control Group: *n* = 104). Five participants were lost to follow-up either before, during, or immediately after treatment (mEHT Group: *n* = 3, Control Group: *n* = 2) and were excluded from OS and DFS analyses. Four participants were lost to follow-up after treatment and could not be contacted (mEHT: one lost to follow-up at six-, nine-, and 18 months post-treatment; Control group: one lost to follow-up at 24 months post-treatment). These participants were excluded from the survival analysis, and their last recorded disease status and follow-up date were used for the DFS analyses (Figure 1). There were no significant differences in participant characteristics and treatment characteristics between the mEHT and Control groups (Table 2 and Table 3). Two thirds of the participants had FIGO Stage III disease and half of all the participants were HIV-positive with more than two thirds of the HIV-participants in the under 50 years old age group. The median age was 50.1 (27.3–74.8), and 79% of participants were unemployed. The median RT dose received was 74 Gy (range: 2–74) and the average dose of cisplatin received was 131 mg/m^2^ per participant, with 12% of participants not receiving any cisplatin. In the mEHT Group, 97% of participants received 80% (8/10), or more of the prescribed mEHT treatments, with only 2% receiving 20% (2/10) or less of the prescribed mEHT treatments. All participants with a haemoglobin value < 10 g/dL at enrolment were transfused before treatment.

### 3.2. Two Year Survival

**Figure 2:**
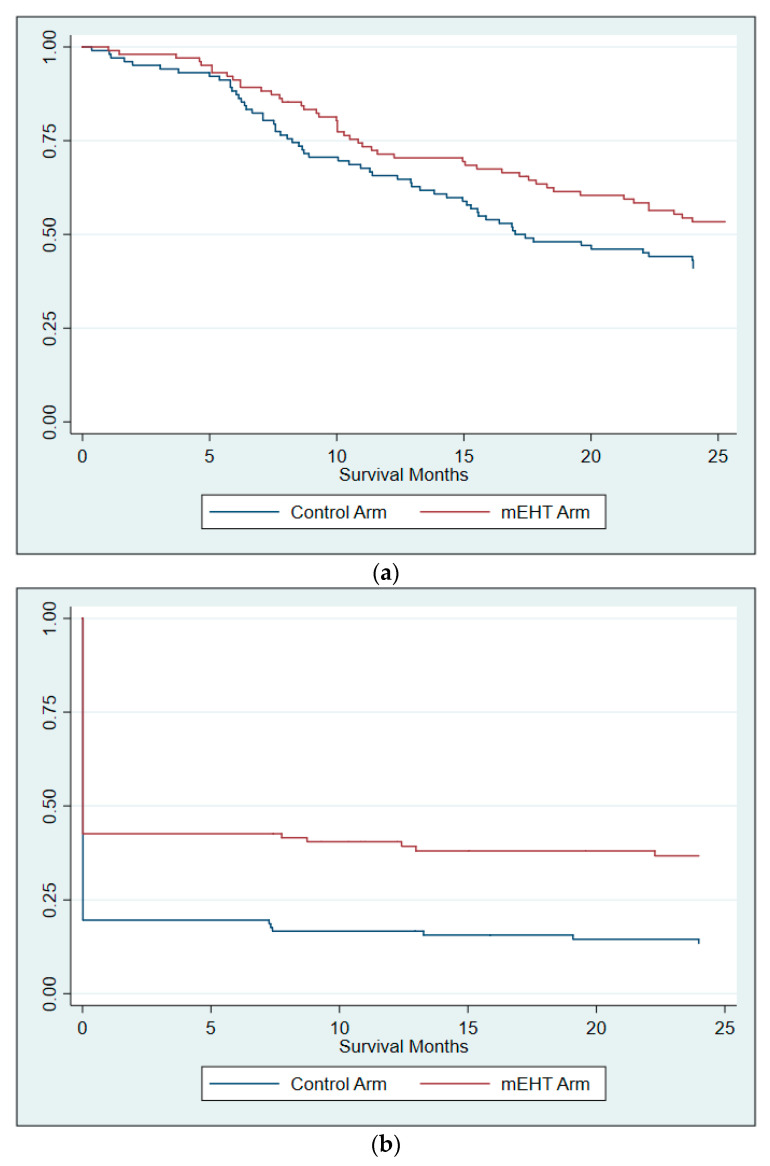
Kaplan–Meier survival curves at two years (**a**) two year overall survival; (**b**) two year disease free survival. The sharp drop of the DFS rates seen early on in 2b is a result of the higher rate of residual disease at six months post-treatment in the Control Group compared to mEHT Group. Participants with residual disease post-treatment were considered to have zero disease free survival days.

Survival data were available for 202 participants at two years post-treatment, (mEHT Group: *n* = 100; Control Group: *n* = 102), of which 53 [53%] and 43 [42%] participants in the mEHT Group and Control Group, respectively, were alive at the last follow-up. The frequency of participants achieving two year OS in the group with LDC at six months post-treatment (42/59 [71.2%]) was significantly higher than those who did not achieve LDC (17/59 [28.8%]; Pearson Chi2: *p <* 0.001). Local Disease Control is a significant predictor of two year OS (OR: 3.8; *p <* 0.001; 95%CI: 2.00–7.34). The risk of death was 30% lower in the mEHT group (HR: 0.70; *p* = 0.074; 95%CI: 0.48–1.03, adjusted for HIV status, age and FIGO stage) (Table 4, Figure 2a)**.**

When considering participants with Stage II and Stage III disease separately, the risk of death within two years post-treatment, adjusted for age, disease stage, and HIV status, was significantly lower in the mEHT participants with Stage III disease compared to the Control participants with Stage III disease (mEHT Group: 34/61 [56%]; Control Group: 27/67 [40%]; HR: 0.61; *p =* 0.047; 95%CI: 0.37–0.99). Age was also a significant predictor of two year OS in the group of participants with Stage III disease (HR: 0.96, *p* = 0.006, 95%CI: 0.94–0.99) (Table 4).

When analysing the sample by treatment arm, age was a significant predictor of two year OS in the mEHT Group (HR: 0.95, *p = 0.001*, 95%CI: 0.93–0.98), but not in the Control Group (HR: 0.98, *p* = 0.181, 95%CI: 0.96–1.01). We subsequently analysed participants according to their age group at the time of randomization (30 years; 30–50 years; >50 years). As there were only three participants younger than 30 years, we combined them with the group of participants between 30 and 50 years. Considering the participants younger than 50 years, and 50 years and older separately, the addition of mEHT had the most significant effect on two year OS in the age group 50 years and above (HR: 0.44, *p =* 0.011, 95%CI: 0.24–0.83).

Two year DFS was seen significantly more frequently in the mEHT Group (36/99 [36.4%]) than in the Control Group (14/102 [13.7%]; *p <* 0.0001), with participants treated with mEHT having 33% less risk of developing a recurrence during the first two years than the Control Group participants (HR: 0.67, 95%CI: 0.48–0.93. *p =* 0.017, adjusted for age, stage, and HIV status) (Table 5, Figure 2b). Participants treated with mEHT had an odds ratio of 3.59 of achieving disease free status at two years (*p <* 0.001; 95%CI: 1.79–7.21) compared to Control Group participants. When evaluated by disease stage, mEHT was not significantly predictive of two year DFS in participants with Stage II disease but remained significant for participants with Stage III disease (Table 5).

### 3.3. Three Year Survival

**Figure 3:**
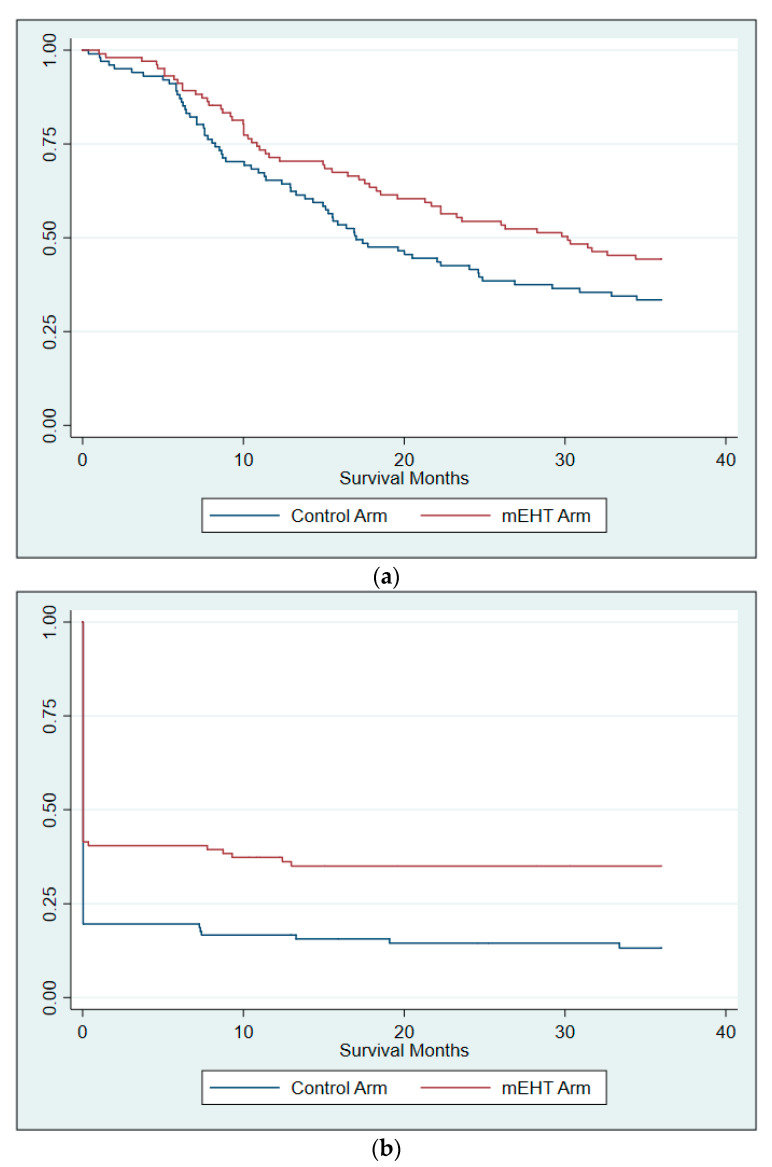
Kaplan–Meier survival curves at three years (**a**) three year overall survival; (**b**) three year disease free survival. The sharp drop off in DFS rates seen early on in 3b is again a result of the high rate of residual disease at six months post treatment.

Three year OS was achieved by 33.7% (34/101) and 44% (44/100) of participants from the Control and mEHT Groups, respectively. The risk of death in the first three years was 28% lower for the participants who received mEHT, although this was not significant (HR: 0.72; 95%CI: 0.51–1.03, *p* = 0.74; adjusted for age, disease stage and HIV status) (Figure 3a), and when considering only the participants with Stage III disease, the risk was significantly lower (38%) in the mEHT group (HR: 0.62; *p =* 0.040; 95%CI: 0.40–0.98, adjusted for age, and HIV status) (Table 6).

The frequency of DFS remained significantly higher in the mEHT Group compared to the Control Group at three years post-treatment (mEHT: 35/99 [35.4%]; Control: 14/102 [13,7%]; Chi-squared: *p* < 0.0001) with an odds ratio of 3.4 of achieving DFS in favour of the mEHT Group (*p =* 0.001; 95%CI: 1.71–6.91) and a hazard ratio of 0.70 (95%CI: 0.51–0.97; *p =* 0.035, adjusted for age, stage and HIV status) (Figure 3b). When evaluated by stage of disease, the significance remained in participants with Stage III disease (Table 7).

### 3.4. Late Toxicity

There was no significant difference in frequencies of reported late toxicity (grouped according to grades I/II and grades III/IV), between the two treatment groups or between the HIV-positive and HIV–negative participants at 9 months, 12 months, 18 months, and 24 months post-treatment. Multivariate Cox proportionate hazards models, including arm, HIV status and cisplatin doses, did not show any significant predictors of grades I/II or grades III/IV late toxicity.

### 3.5. Quality of Life

There were no statistically significant differences in QLQ scores between the two groups at baseline assessment [28]. When comparing the changes in scores from baseline to 24 months between groups, the reduction in pain was significantly higher in the mEHT Group (*p =* 0.0368), cognitive function was significantly improved in the mEHT group (*p =* 0.0044), and participants in the Control Group reported a reduction in role functioning while the mEHT Group participants reported an improvement in role functioning with a significant difference between the two groups (*p =* 0.0172). When assessing the change from baseline to 12 months within each group, there was an improvement in all scales except for role functioning in the mEHT Group, with significant improvements in Global Health Scale, Pain, Fatigue, and Emotional functioning. In the Control Group, there were significant improvements in the Visual Analogue Scale, Global Health Scale, Nausea and Vomiting, and Emotional Functioning, while Physical Functioning, Role Functioning and Cognitive Functioning decreased in the Control Group (Table 8). When assessing the change from baseline to 24 months within each group, the mEHT group reported a significant improved of all scales except for role function (which improved by a score of 9.4), while the Control Group only reported a significant change in five out of 11 scales, with a negative change in cognitive function (Table 9).

### 3.6. The Abscopal Effect

We previously reported on an increased frequency of an abscopal effect seen in the mEHT participants at six months post-treatment [29]. The three year follow-up of these participants shows that 10 of the 14 mEHT participants with an abscopal effect were disease free at three years post-treatment, and three participants were deceased, two of whom were disease free at death (cause of death renal failure, DFS days 335 and 596), and one whom was disease free at the last follow-up with an unknown cause of death after 860 days. Of the three participants in the Control Group who had an abscopal response, two achieved three year DFS and one demised after 483 days, due to renal failure. The disease pattern and description of these participants are detailed in our previous paper on the abscopal effect seen at six months post-treatment [29].

### 3.7. Cost Effectiveness Analysis

**Figure 4:**
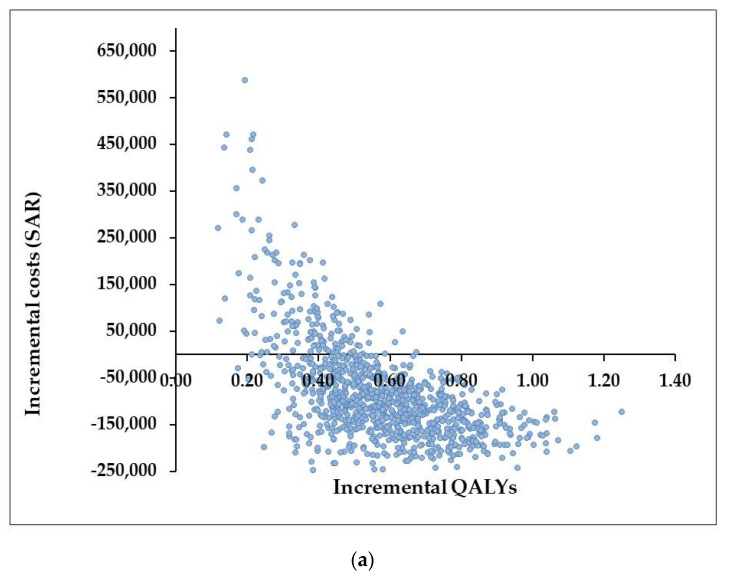

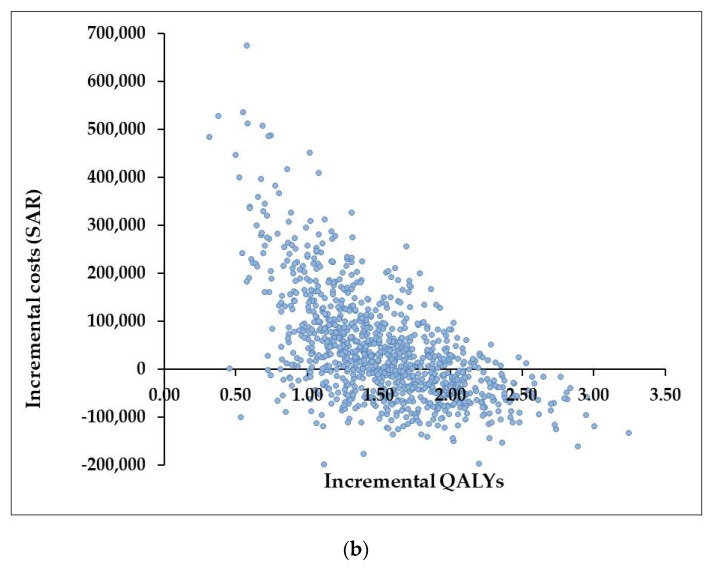
Incremental cost-effectiveness ratio (ICER) plane (**a**) government healthcare model; (**b**) private healthcare model. The Cost Effectiveness Analysis was done for both a Government-funded and a privately-funded healthcare model, for the same duration (three years), assuming the same health effects, with the only difference being the input costs. In the Government-funded healthcare model, the QALYs range from 0–1.4, with incremental costs mainly seen in the 4th Quadrant, showing improved clinical benefits and lower costs per QALY with the addition of mEHT. In the Privately funded healthcare model, the QALYs range from 0–3.5 with incremental costs falling in the lower portion of the 1st quadrant and the upper portion of the 4th quadrant, implying a clinical benefit with a high probability of cost saving with the addition of mEHT to chemoradiotherapy

The addition of mEHT to CRT increases the efficacy of the oncology treatments; however, it also increases the initial input costs. The base case CEA showed that the addition of mEHT to CRT dominated the model, compared to CRT alone, making the combined treatment (mEHT + CRT) less costly and more effective, from the perspective of both government and private healthcare funders. This result is driven by the difference in DFS and is due to the high costs of recurrent and progressive disease. This model did not use a societal costing perspective, which incorporates productivity-loss costs as well as dying costs, especially before retirement age. The incremental cost-effectiveness ratio (ICER) plane shows that CRT + mEHT produces more health effects at a lower cost over three years, in the government and private healthcare model, per disease free cycle (a half year lived in perfect health) (Figure 4). The probability that mEHT + CRT is cost-effective compared with CRT alone is about 82.2% in the government healthcare model and 77.7% in the private healthcare model, at no additional cost. The QALYs are summarised in Table 10.

## 4. Discussion

The results from this study show a significant improvement in two and three year DFS with the addition of mEHT to CRT protocols for LACC, without any significant changes in late toxicity. This follows our previous paper describing the improvement in LDC with the addition of mEHT to CRT. The strict criteria for LDC evaluation is one of the strengths of the study. Evaluation of LDC was based on pre- and post-treatment 18^F^-FDG PET/CT scans, examinations, and fine needle aspiration if indicated. Local disease control was considered a failure if any disease was confirmed in the pelvis [27]. We previously described the safety of mEHT in our paper on early toxicity, and reported high compliance rates to mEHT treatments in our high risk population. Other strengths of the study include the low variability in patient and treatment characteristics between the groups, the strict control between the groups, and the low number of participants lost to follow-up, even in the resource-constrained setting.

Our sample included HIV-positive participants, who are expected to have worse outcomes [36,37,38], and overweight participants [28]. Radiobiological data have previously suggested that HIV-positive patients may be more radiosensitive, and may therefore be at risk of increased toxicity from RT [39,40]. The evaluation of the early and late toxicity associated with RT combined with mEHT as a radiosensitiser in HIV-positive patients is therefore important in our setting where around 50% of LACC patients are HIV-positive. Heating pelvic tumours using capacitive HT techniques carries a high risk of adipose burns, especially when the treatment area includes a layer of adipose tissue thicker than 1.5 cm [41,42]. The safety demonstrated by mEHT for the management of cervical cancer, even in participants with above average BMIs, alongside the efficacy, indicates that mEHT is able to effectively and safely target deep tumours that would otherwise be difficult to treat using conventional capacitive HT. Factors which may contribute to the improved safety and efficacy of mEHT include the lower power output of mEHT (maximum of 130 W in our study), compared to other capacitive HT devices, the non-thermal effects [14] or field effects [16,43], and the AM of the RF waves in mEHT, which appears to contribute to the improved selectivity and enhanced effects in the tumour [18,25].

We initially estimated a reduction in two year mortality of 50% in order to achieve a power of 90% based on our sample size. While two year OS rates were not significantly improved, the reduction in disease recurrence at two years in the mEHT group was significant and was more than 50% (36.4% DFS in the mEHT Group and only 13.7% DFS in the Control Group), giving a statistical power of >90% for the DFS assessment. The effect of mEHT on outcomes was seen more significantly in the two year and three year DFS analyses than in the OS analyses, and the significance remained in both the HIV-positive and -negative participants and when considering participants based on age category. However, the significance was lost when considering only the participants with Stage II disease. In the OS analyses, the significance of the effects of mEHT on outcomes was less consistent. This may be a result of the inclusion of non-cancer related deaths in the OS analysis, which likely masked the effects of mEHT in the OS analyses. In our sample, the majority of the HIV-positive participants were younger than 50 years, and this may contribute to the improved OS outcomes seen in participants over the age of 50 years, compared to those younger than 50 years. This suggests that, while mEHT still improves the OS of HIV-positive participants, the effect is higher in HIV-negative participants as seen in the older group containing mostly HIV-negative women. In the group of participants who were 50 years and older, mEHT was a consistently significant predictor of DFS, regardless of HIV-status.

A limitation of the study is the substandard RT and BT administered as a result of a lack of sophisticated imaging and planning techniques in our setting, compared to developed settings. Due to resource constraints, the standard of care weekly cisplatin schedule was also not prescribed. Other limiting factors related to resource constraints include time to start EBRT, time to complete RT, and time between treatment completion and ^18^F-FDG PET/CT scans. Delays were most frequently attributed to technical problems, machine down-time, and source supply problems (in the case of the ^18^F-FDG PET/CT scans), as previously reported [27]. Another limitation of the study is the apparent high rate of under-staging of the patients using clinical staging techniques. The participants were all staged according to the recommended FIGO staging guidelines from 2014, and the institutional protocols at the time, using a chest X-ray, abdomino-pelvic ultrasound, and examination. The FIGO staging system was revised in 2018 to include more sophisticated imaging techniques which are able to include lymph node involvement and to improve the accuracy of the staging. The earlier FIGO staging criteria resulted in up to 40% of stage IB-IIIB cases being under diagnosed and as many as 64% of stage IIIB cases being over-diagnosed [44]. Funding was obtained for the addition of ^18^F-FDG PET/CT scans pre-treatment and six months post-treatment to assess clinical response to treatment. The ^18^F-FDG PET/CT scans were therefore not used for staging purposes in our study; however, participants with visceral and bone metastases and bilateral hydronephrosis on the ^18^F-FDG PET/CT scans were still excluded as they required a change in the treatment protocol. The pre-treatment ^18^F-FDG PET/CT scans indicated that more than half of the patients were in stage IVB disease, as seen by the high number of patients with extra-pelvic nodal involvement and local invasion of the bladder and rectum that was not detected during the routine clinical staging procedures. In a sub-group analysis of these participants, it was noted that there was complete metabolic resolution of all diseases, local and distant, in around a quarter of those who received mEHT. This suggests that mEHT may potentiate the abscopal effect induced by ionising radiation. This also provided an opportunity to assess the systemic effects of mEHT. The previously reported abscopal results [29], combined with the long term follow-up of the abscopal response reported in this paper, suggest that the preclinical immunological effects, observed in response to the administration of mEHT [45,46,47], could have clinical benefits in the management of systemic disease as well as local disease. If we consider that Stage IVB disease is generally considered incurable, then a disease free status in the participants with extra-pelvic disease at three years of 24.5% in the mEHT group compared to the 5.6% in the control group, even with sub-optimal RT delivery, is a significant and important outcome.

Only one Phase III study has investigated CRT with/without classical HT (using capacitive HT), for the management LACC, and they reported an improvement in five year DFS from 60.6% (95%CI, 45.3–72.9%) to 70.8% (95%CI, 55.5–81.7%), although the difference was not significant (HR: 0.517, 95%CI, 0.251–1.065, *p* = 0.073) [48]. While results from Phase III studies on RT with/without classical HT are positive, they are not comparable to our study, due to the differences in HT techniques and treatment protocols. Classical HT requires a substantial increase in local temperature in order to slow down DNA repair and induce tumour cell killing [12], and thermo-monitoring is a critical safety and efficiency measure [10], while mEHT aims to improve perfusion and support an immune response to the tumours [49], with a mild temperature increase, and without the need for thermo-monitoring as a measure of safety and efficiency.

The substantial improvements in quality of life are an important result to consider as prolonged life is not always associated with quality of life in cancer patients. The adverse effects from oncology treatments can negatively impact the quality of life even in patients who are disease free, while persistent and recurrent disease are often considered to be poor predators of quality of life. An increase in life expectancy, together with a decreased quality of life and increased costs of treatment for adverse effects and persistent/recurrent disease, can place additional burden on the healthcare system. The CEA performed confirms that the improvement in quality of life, and improvement in DFS, not only benefits the patients and the community, but also has the potential to reduce the economic burden of the disease in both private and public healthcare settings.

While it is unclear how much of an effect mEHT as a radiosensitiser would have when added to optimal RT and BT delivery for cervical cancer, it is encouraging to see such a large improvement in two and three year DFS with the addition of mEHT, even in sub-optimal conditions and in our high-risk population. There is still room for improvement in five year OS rates in cervical cancer patients with stage III and IV disease globally, even with sophisticated RT techniques, and a safe and effective radiosensitiser, such as mEHT, may still be a beneficial adjunct to RT in optimal settings. The continued monitoring of participants in the reported study will provide more insight into the effects of mEHT on five year survival. Modulated electro-hyperthermia is a feasible addition to LACC treatment protocols to improve outcomes, especially in settings in which sophisticated imaging and RT technologies are not accessible.

## 5. Conclusions

Modulated electro-hyperthermia enhances outcomes of LACC patients when added to CRT, without increasing the toxicity profile of treatments. The associated improvement in quality of life along with the reduction in healthcare costs makes this intervention a feasible and effective adjunct to CRT for the management of LACC. The addition of mEHT improved LDC and DFS in our sample, without additional toxicity, and with improved role functioning of the patients, benefiting both the patients, the community, and the already-strained healthcare system. Modulated electro-hyperthermia could therefore be considered as an adjunct to CRT, especially in resource-constrained settings and for cervical cancer patients with advanced disease. The five year follow-up results and detailed CEA will provide further insight into the long term benefits of mEHT as an adjunct to CRT. Further investigations into the immunological effects of mEHT could assist in the long-term goal of shifting RT from a local treatment, to a systemic treatment when combined with mEHT, offering additional options for patients with metastatic disease. Studies on the systemic effects of mEHT, as well as studies with the aim of better understanding the thermal and non-thermal effects of mEHT, are likely to shed more light on the mechanisms of action and further improve the application and recommendations for the use of mEHT in a clinical setting.

## Figures and Tables

**Table 1:** Summary of the local disease control results at six months post-treatment [27].

210 Randomised Participants	Total		mEHT		Control		Chi Squared
	*n*	%	*n*	%	*n*	%	
Eligible for analysis	202	96.2%	101	50.0%	101	50.0%	
Alive at six months post-treatment	171	84.7%	88	87.1%	83	82.2%	*p* = 0.329
LDC achieved	60	29.7%	40	45.5%	20	24.1%	*p =* 0.003
LDFS achieved in those who survived six months post-treatment	59	29.2%	39	38.6%	20	19.8%	*p =* 0.003

Abbreviations: LDC: Local Disease Control; LDFS: Local Disease Free Survival; mEHT: Modulated electro-hyperthermia.

**Table 2:** Participant characteristics.

Participant Characteristic		mEHT		Control		*p*-Value
		106	(50.5%)	104	(49.5%)	
HIV Status	Positive	52	(49.1%)	55	(52.9%)	*p* = 0.579
	Negative	54	(50.9%)	49	(47.1%)	
Age Group	<50 years	52	(49.1%)	46	(44.2%)	*p* = 0.483
	≥50 years	54	(50.9%)	58	(55.8%)	
ECOG	0	3	(2.8%)	7	(6.7%)	*p* = 0.184
	1	103	(97.2%)	97	(93.3%)	
Race	African	98	(92.5%)	97	(93.3%)	*p* = 0.335
	Caucasian	4	(3.8%)	1	(1.0%)	
	Indian	0	(0.0%)	0	(0.0%)	
	Asian	0	(0.0%)	0	(0.0%)	
	Mixed Race	4	(3.8%)	6	(5.8%)	
Education	Primary	45	(43.3%)	50	(49.0%)	*p* = 0.334
	Secondary	55	(52.9%)	51	(50.0%)	
	Tertiary	4	(3.8%)	1	(1.0%)	
Employment	Unemployed	83	(78.3%)	82	(78.8%)	*p* = 0.923
	Employed	23	(21.7%)	22	(21.2%)	
FIGO	IIB	40	(37.7%)	36	(34.6%)	*p* = 0.895
Staging	IIIA	1	(0.9%)	1	(1.0%)	
	IIIB	65	(61.3%)	67	(64.4%)	
Histological Grade	1	7	(6.9%)	4	(4.1%)	*p* = 0.759
	2	70	(69.3%)	67	(69.1%)	
	3	24	(23.8%)	26	(26.8%)	
Tumour Dimensions (cm)	Median	7		7.1		*p* = 0.1429
	Min	2.7		1.8		
	Max	11.7		14.87		
Tumour SUV	Median	18.07		19.26		*p* = 0.7769
	Min	7.01		6.07		
	Max	63.25		97		
HB (g/dL)	Median	10.9		11		*p* = 0.9424
	Min	5.7		5.2		
	Max	16.2		16.2		
Age	Median	49.2		50.6		*p* = 0.3665
	Min	27.3		29.2		
	Max	70.8		74.8		
BMI	Median	27		26.5		*p* = 0.3883
	Min	15		15		
	Max	49		41.7		

Abbreviations: BMI: Body Mass Index; ECOG: Eastern Cooperative Oncology Group; FIGO: Fédération Internationale de Gynécologie et d’Obstétrique; HB: Haemoglobin; HIV: Human Immunodeficiency Virus; mEHT: Modulated Electro-Hyperthermia; SUV: Standard Uptake Value.

**Table 3:** Treatment characteristics.

Treatment		mEHT		Control		*p*-Value
Characteristics		106	(50.5%)	104	(49.5%)	
No of HDR BT doses	0	0	(0.0%)	0	(0.0%)	*p* = 0.223
	1	0	(0.0%)	2	(2.0%)	
	2	3	(2.9%)	1	(1.0%)	
	3	101	(97.1%)	99	(97.1%)	
No of Cisplatin Doses	0	14	(13.6%)	11	(10.7%)	*p* = 0.727
	1	42	(40.8%)	47	(45.6%)	
	2	47	(45.6%)	45	(43.7%)	
Total RT Dose	Median	74		74		*p* = 0.6133
	Min	20		2		
	Max	74		74		
Days between enrolment and Treatment	Median	37		37		*p* = 0.2241
	Min	18		21		
	Max	79		104		
No of mEHT doses	Median	10				
	Min	1				
	Max	10				

Abbreviations: HDR BT: High Dose Rate Brachytherapy; mEHT: Modulated Electro-Hyperthermia; RT: Radiotherapy.

**Table 4:** Multivariable Cox proportional hazards model for two year overall survival.

**Overall**	**HR**	***p*-Value**	**[95%CI]**
mEHT	0.70	0.074	0.48–1.03
HIV-negative	0.82	0.328	0.54–1.23
Age at Enrolment	0.97	*0.007*	0.95–0.99
FIGO Stage III	1.01	0.785	0.71–1.57
**FIGO Stage II**	**HR**	***p*-Value**	**[95%CI]**
mEHT	0.88	0.677	0.47–1.64
HIV-negative	0.73	0.342	0.37–1.41
Age at Enrolment	0.99	0.401	0.96–1.02
**FIGO Stage III**	**HR**	***p*-Value**	**[95%CI]**
mEHT	0.61	0.047	0.37–0.99
HIV-negative	0.90	0.699	0.54–1.52
Age at Enrolment	0.96	0.006	0.94–0.99

Abbreviations: FIGO: Fédération Internationale de Gynécologie et d’Obstétrique; HIV: Human Immunodeficiency Virus; HR: Hazard Ratio; mEHT: Modulated Electro-Hyperthermia.

**Table 5:** Multivariable Cox proportional hazards model for two year disease free survival.

**Overall**	**HR**	***p*-Value**	**[95%CI]**
mEHT	0.67	0.017	0.48–0.93
HIV-negative	0.99	0.257	0.72–1.48
Age at Enrolment	0.99	0.257	0.97–1.01
FIGO Stage III	0.99	0.944	0.79–1.38
**FIGO Stage II**	**HR**	***p*-Value**	**[95%CI]**
mEHT	0.77	0.342	0.45–1.32
HIV-negative	1.18	0.569	0.66–2.01
Age at Enrolment	0.99	0.601	0.97–1.02
**FIGO Stage III**	**HR**	***p*-Value**	**[95%CI]**
mEHT	0.62	0.025	0.41–0.94
HIV-negative	0.98	0.915	0.97–1.01
Age at Enrolment	0.99	0.301	0.97–1.01

Abbreviations: FIGO: Fédération Internationale de Gynécologie et d’Obstétrique; HIV: Human Immunodeficiency Virus; HR: Hazard Ratio; mEHT: Modulated Electro-Hyperthermia.

**Table 6:** Multivariable Cox proportional hazards model for three year overall survival.

**Overall**	**HR**	***p*-Value**	**[95%CI]**
mEHT	0.72	0.074	0.51–1.03
HIV-negative	0.84	0.366	0.58–1.23
Age at Enrolment	0.98	*0.019*	0.96–1.00
FIGO Stage	1.10	0.619	0.76–1.59
**FIGO Stage II**	**HR**	***p*-Value**	**[95%CI]**
mEHT	0.91	0.748	0.51–1.64
HIV-negative	0.75	0.365	0.40–1.40
Age at Enrolment	0.99	0.468	0.96–1.02
**FIGO Stage III**	**HR**	***p*-Value**	**[95%CI]**
mEHT	0.62	*0.040*	0.40–0.98
HIV-negative	0.93	0.777	0.58–1.50
Age at Enrolment	0.97	*0.018*	0.95–0.99

Abbreviations: FIGO: Fédération Internationale de Gynécologie et d’Obstétrique; HIV: Human Immunodeficiency Virus; HR: Hazard Ratio; mEHT: Modulated Electro-Hyperthermia.

**Table 7:** Multivariable Cox proportional hazards model for three year disease free survival.

**Overall**	**HR**	***p*-Value**	**[95%CI]**
mEHT	0.70	0.035	0.51–0.98
HIV-negative	1.05	0.786	0.74–1.50
Age at Enrolment	0.99	0.240	0.97–1.01
FIGO Stage	0.98	0.913	0.70–1.37
**FIGO Stage II**	**HR**	***p*-Value**	**[95%CI]**
mEHT	0.78	0.357	0.46–1.33
HIV-negative	1.20	0.538	0.68-2.11
Age at Enrolment	0.99	0.582	0.97–1.02
**FIGO Stage III**	**HR**	***p*-Value**	**[95%CI]**
mEHT	0.66	0.040	0.43–0.98
HIV-negative	0.98	0.932	0.62–1.55
Age at Enrolment	0.99	*0.278*	0.97–1.01

Abbreviations: FIGO: Fédération Internationale de Gynécologie et d’Obstétrique; HIV: Human Immunodeficiency Virus; HR: Hazard Ratio; mEHT: Modulated Electro-Hyperthermia.

**Table 8:** Mean change in scores from baseline to 12 months in the mEHT and Control Group.

12 Months	mEHT				Control			
	Mean	SD	95%CI	*p*-Value	Mean	SD	95%CI	*p*-Value
Visual Analogue	5.4	31.6	−2.9 to 13.8	*p =* 0.1961	9.7	29.8	2.1 to 17.3	*p =* 0.0133
Global Health	10.2	34.3	1.2 to 19.2	*p =* 0.0275	13.8	36.3	4.4 to 23.1	*p =* 0.0047
Financial Burden	−7.1	50.7	−207 to 6.4	*p =* 0.2967	−6.1	48.0	−19.1 to 7.0	*p =* 0.3537
Symptom Scales								
Pain Reduction	−18.4	37.3	−28.2 to −8.6	*p =* 0.0004	−6.3	40.2	−16.6 to 4.0	*p =* 0.2264
Nausea/Vomiting	−5.5	23.4	−11.6 to 0.7	*p =* 0.0815	−6.5	19.1	−11.4 to −1.7	*p =* 0.0094
Fatigue reduction	−9.4	31.0	−17.5 to −1.2	*p =* 0.0247	−1.3	40.5	−11.6 to 9.1	*p =* 0.8065
Functional Scales								
Social	5.5	46.9	−6.9 to 17.8	*p =* 0.3787	2.6	55.2	−12.0 to 17.3	*p =* 0.7201
Cognitive	7.5	31.9	−0.9 to 15.9	*p =* 0.0795	−1.1	34.0	−10.1 to 7.3	*p =* 0.7542
Emotional	9.8	31.9	1.4 to 18.2	*p =* 0.0233	13.4	39.9	3.2 to 23.6	*p =* 0.0111
Role	−3.2	40.9	−13.9 to 7.6	*p =* 0.5583	−4.9	40.0	−15.2 to 5.3	*p =* 0.3401
Physical	2.3	29.9	−5.6 to 10.2	*p =* 0.5599	−4.0	27.7	−11.2 to 3.1	*p =* 0.2594

Abbreviations: CI: Confidence Interval; mEHT: Modulated Electro-Hyperthermia; SD: Standard Deviation.

**Table 9:** Mean change in scores from baseline to 24 months in the mEHT and Control Group.

	mEHT				Control			
	Mean	SD	95%CI	*p*-Value	Mean	SD	95%CI	*p*-Value
Visual Analogue	25.1	21.5	16.6 to 33.6	*p <* 0.0001	15.6	31.9	2.9 to 28.2	*p =* 0.0176
Global Health	23.2	31.7	11.7 to 35.6	*p =* 0.0002	17.3	29.1	6.0 to 28.6	*p =* 0.0041
Financial Burden	−26.1	60.9	−48.0 to 4.1	*p =* 0.0216	−16.7	46.7	−34.8 to 1.4	*p =* 0.0698
Symptom Scales								
Pain Reduction	−34.4	32.8	−46.2 to −22.6	*p =* 0.0001	−15.5	35.7	−29.3 to −16	*p =* 0.0298
Nausea/Vomiting	−13.0	27.7	−23.0 to −3.0	*p =* 0.0122	−1.2	18.7	−8.4 to 6.1	*p =* 0.7383
Fatigue reduction	−18.4	27.9	−28.5 to −8.4	*p =* 0.0008	−10.7	34.0	−23.9 to 2.4	*p =* 0.1071
Functional Scales								
Social	12.0	31.2	0.7 to 23.2	*p =* 0.0375	17.3	41.7	1.1 to 33.4	*p =* 0.0373
Cognitive	19.8	33.2	7.8 to 31.6	*p =* 0.0020	−4.2	28.9	−15.4 to 7.0	*p =* 0.4523
Emotional	27.3	30.3	16.4 to 38.3	*p <* 0.0001	17.9	34.2	4.6 to 31.1	*p =* 0.0101
Role Function	9.4	35.1	−3.3 to 22.1	*p =* 0.1415	7.1	35.0	6.4 to 20.7	*p =* 0.2893
Physical	11.7	21.2	4.0 to ‘9.3	*p =* 0.0040	2.6	27.2	−7.9 to 13.2	*p =* 0.6150

Abbreviations: CI: Confidence Interval; mEHT: Modulated Electro-Hyperthermia; SD: Standard Deviation.

**Table 10:** Quality adjusted life year data for private and government healthcare CEA models.

Perspective	Treatment	Cost in ZAR	QALYs Gained *	Incremental Cost	Incremental QALYs *	ICER
Government	mEHT	412,433.37	4.84			
	CRT	449,290.02	4.60	36,836.65	−0.24	Dominated
Private payer	mEHT	579,998.97	4.84			
	CRT	617,421.79	4.60	37,422.82	−0.24	Dominated

* QALYs gained in the two perspectives are the same since assumptions for health effects were the same. The only differences in the model inputs were the costs.

## Data Availability

The data presented in this study will be made openly available in (repository name e.g., FigShare) upon acceptance of the paper for review.
